# Short-term air pollution exposure aggravates Parkinson’s disease in a population-based cohort

**DOI:** 10.1038/srep44741

**Published:** 2017-03-16

**Authors:** Hyewon Lee, Woojae Myung, Doh Kwan Kim, Satbyul Estella Kim, Clara Tammy Kim, Ho Kim

**Affiliations:** 1 Graduate School of Public Health, Seoul National University, South Korea; 2 Department of Psychiatry, CHA Bundang Medical Center, CHA University, Bundang-gu Seongnam-si, Gyeonggi-do, South Korea; 3 Department of Psychiatry, Samsung Medical Center, Sungkyunkwan University School of Medicine, Seoul, South Korea

## Abstract

Increasing experimental evidence has suggested air pollution as new risk factor for neurological disease. Although long-term exposure is reportedly related to neurological disease, information on association with short-term exposure is scarce. We examined the association of short-term exposure to particles <2.5 μm (PM_2.5_), nitrogen dioxide (NO_2_), sulfur dioxide (SO_2_), ozone (O_3_), and carbon monoxide (CO) with PD aggravation in Seoul from the National Health Insurance Service–National Sample Cohort, Korea during 2002–2013. PD aggravation cases were defined as emergency hospital admissions for primarily diagnosed PD and analyzed with a case-crossover analysis, designed for rare acute outcomes. Pollutants concentrations on case and control days were compared and effect modifications were explored. A unit increase in 8-day moving average of concentrations was significantly associated with PD aggravation. The association was consistent for PM_2.5_ (odds ratio [95% confidence interval]: 1.61 [1.14–2.29] per 10 μg/m^3^), NO_2_ (2.35 [1.39–3.97] per 10 ppb), SO_2_ (1.54 [1.11–2.14] per 1 ppb), and CO (1.46 [1.05–2.04] per 0.1 ppm). The associations were stronger in women, patients aged 65–74 years, and cold season, but not significant. In conclusion, short-term air pollution exposure increased risk of PD aggravation, and may cause neurological disease progression in humans.

Parkinson’s disease (PD) is the second-most prevalent neurodegenerative disease, following Alzheimer’s disease (AD) worldwide; the socio-economic burden attributable to PD is expected to increase with the aging of the population^1^,^2^. In South Korea, the number of PD patients has been rapidly increasing (approximately 24,300 incidence cases between 2010 and 2014), and the total medical expenses for PD has more than doubled (to USD 222 million in 2014). Given the progression to a super-aged society, the number of PD patients in South Korea is expected to increase to 87–93 million by 2030^3^.

PD is caused by the loss of dopamine-generating cells in the substantia nigra^4^; however, the exact pathogenesis remains unclear^2^. Recently, neuro-inflammation and oxidative stress have been increasingly considered causal factors in the pathology of central nervous system (CNS) diseases^5^. Although various environmental factors may be involved, air pollution has been identified as the most pervasive factor inducing inflammation and oxidative stress^6^. Air pollution has been consistently associated with cardiovascular and respiratory diseases^7^,^8^, and is now considered an emerging risk factor for neurological diseases. Recent experimental studies have shown that air pollutants cause neuro-inflammation, CNS oxidative stress, dopamine neuron damage, blood-brain barrier (BBB) damage, and cerebrovascular impairment^6^,^9^, which indicate potential biological pathways for neurological diseases. Considering the increasing experimental evidence linking air pollution and neurological damage, epidemiological studies have been conducted on the association between long-term exposure to air pollution and neurological diseases. Decreased cognitive function in humans has been related to increasing annual concentrations of black carbon (BC)^10^, particles less than 10 μm in aerodynamic diameter (PM_10_), and particles less than 2.5 μm in aerodynamic diameter (PM_2.5_)^11^. PD incidence has been associated with annual increases in airborne metal concentrations^12^, very long-term exposure (over 20 years) to nitrogen dioxide (NO_2_)^13^, and annual increases in PM_10_ and PM_2.5_ among female never smokers^14^; AD incidence has been associated with increasing annual exposure to nitrogen oxides (NO_x_)^15^, NO_2_, and carbon monoxide (CO)^16^. A recent study focusing on PM_2.5_ involvement in neurological disease progression found that long-term PM_2.5_ exposure had significant effects on hospitalizations for dementia, AD, and PD^17^.

However, information on the association between short-term air pollution exposure (for days or weeks) and neurological diseases in humans is scarce, although short-term exposure to air pollution has been considered to aggravate neurological function. In the early 1970 s, Lewis^18^ reported that mental efficiency in adults decreased after the breathing of polluted air from the streets in London. Recently, Wellenius *et al*.^19^ found that increasing mean PM_2.5_ concentrations during 1–28 days before evaluation was associated with elevated cerebral vascular residence and reduced cerebral blood flow velocity in a community-dwelling senior cohort. Moreover, Zanobetti *et al*.^20^ reported that the average PM_2.5_ concentrations over the 2 days before evaluation was related to an increased risk of hospitalization for PD. Here, we examine the effects of short-term exposure to 5 air pollutants on the aggravation of PD, defined as cases of emergency hospital admission that were primarily diagnosed with PD, using a time-stratified case-crossover design in Seoul, the largest metropolitan city, from a population-based cohort in South Korea.

## Results

### Confirmation of PD cases and air pollution

During 2002–2013, we identified 77 emergency admission cases with primarily diagnosed PD and 314 emergency admission cases with PD as a primary or an accessory diagnosis. Female patients (56%) and patients aged ≥75 years (53%) were predominant among the PD cases, and the season-specific case counts were similar (Table 1). Dementia, diabetes, and cerebral infarction were the most prevalent comorbidities among the primary PD cases. While the 2-day moving average (lag0–2) concentrations of the pollutants were similar on case and control days, the 8-day moving average (lag0–7) concentrations were higher on case days than on control days, except for O_3_ (Supplementary Table 1). The difference in the lag0–7 concentrations between the case and control days ranged from 0.01 ppm for CO to 2.3 μg/m^3^ for PM_2.5_ among the cases who were primarily diagnosed with PD, and from 0 ppm for CO to 1.1 μg/m^3^ for PM_2.5_ among the cases with a primary or an accessory diagnosis of PD. All the air pollutants were highly inter-correlated with each other (correlation coefficient *r*: 0.54–0.76), except for O_3_ (*r*: 0–0.44), and were not or lowly correlated with the weather variables (*r*: 0–0.47; Supplementary Table 2).

### Short-term association between air pollution and PD aggravation

**Figure 1:**
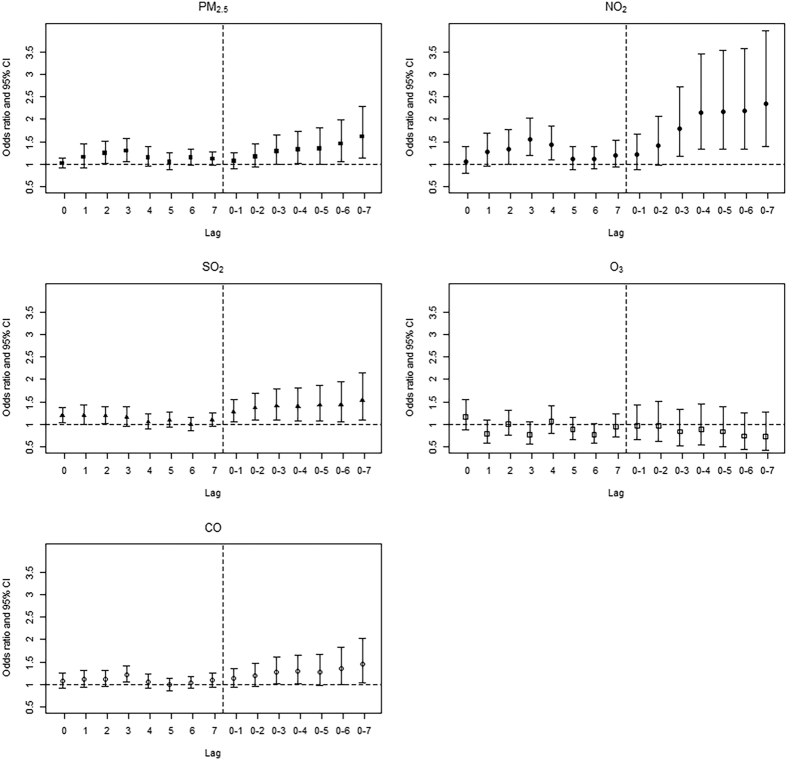
Odds ratios of Parkinson’s disease aggravation associated with a unit^a^ increase in the concentrations of 5 air pollutants with various lag structures (single lag on the same day [lag0] and on the previous 1–7 days [lag1–lag7], as well as moving average lag on the same day plus 1 day before [lag0–1] to 7 days before [lag0–7]). ^a^Units are 10 μg/m^3^ for PM_10_; 10 ppb for NO_2_ and O_3_; 1 ppb for SO_2_; and 0.1 ppm for CO.

Figure 1 shows the odds ratios (ORs) of PD aggravation associated with a unit increase in the concentrations of the 5 air pollutants, with various lag structures; units are 10 μg/m^3^ for PM_10_; 10 ppb for NO_2_ and O_3_; 1 ppb for SO_2_; and 0.1 ppm for CO. We observed significant associations for all air pollutants except for O_3_, and the estimated effects for each pollutant showed a similar lag pattern—i.e., a significant effect for lag3 concentrations (OR [95% CI] for PM_2.5_: 1.29 [1.06–1.57], NO_2_: 1.55 [1.19–2.03], CO: 1.22 [1.05–1.41]) for the single lag structure, and the largest significant effect for lag0–7 concentrations (OR [95% CI] for PM_2.5_: 1.61 [1.14–2.29], NO_2_: 2.35 [1.39–3.97], SO_2_: 1.54 [1.11–2.14], CO: 1.46 [1.05–2.04]) for the moving average lag structure. Based on these results, the lag0–7 concentrations of all the pollutants were selected for further analyses, except for O_3_ (same-day [lag0] concentrations). The ORs of emergency admission for PD including accessory diagnoses were smaller than those for PD aggravation (Supplementary Fig. 2). In the assessment of the possible non-linear associations, we did not find evidence of non-linearity; testing for linearity between short-term air pollution exposure and PD aggravation gave χ^2^ = 0.06, df = 1, P = 0.80 for PM_2.5_, χ^2^ = 1.63, df = 1, P = 0.20 for NO_2_, χ^2^ = 0.15, df = 1, P = 0.70 for SO_2_, χ^2^ = 0.30, df = 1, P = 0.59 for O_3_, and χ^2^ = 0.52, df = 1, P = 0.47 for CO (Supplementary Fig. 3).

### Effect modification by sex, age, and season

Table 2 presents the sex-, age-, and season-specific associations between air pollution and PD aggravation, estimated using interaction terms. In the sex-specific analysis, women showed slightly stronger associations (OR [95% CI] for PM_2.5_: 1.66 [1.10–2.50], NO_2_: 2.15 [1.16–3.99], SO_2_: 1.64 [1.02–2.63], CO: 2.65 [1.05–6.68]) than men (OR [95% CI] for PM_2.5_: 1.53 [0.86–2.72], NO_2_: 2.81 [1.17–6.76], SO_2_: 1.46 [0.94–2.25], CO: 1.34 [0.83–2.15]). Among the different age groups (<65, 65–74, and ≥75 years), the 65–74-year age group generally showed stronger associations (OR [95% CI] for PM_2.5_: 1.95 [1.14–3.32], NO_2_: 2.62 [1.27–5.4], CO: 1.74 [1.04–2.91]) than the other age groups. With regard to the season-specific results, all air pollutants, except for O_3_, showed stronger association with PD in the cool season (OR [95% CI] for PM_2.5_: 1.83 [1.17–2.87], NO_2_: 2.77 [1.38–5.57], SO_2_: 1.68 [1.13–2.51], CO: 1.48 [1.02–2.16]); however, these differences were not significant (*p* interaction = 0.23–0.99). The group-specific results using single-lag concentrations also showed consistent results except in a case of a sex-specific result for SO_2_, which revealed a significantly stronger association in women (*p* interaction = 0.02) (Supplementary Table 3).

### Robustness of the air pollution effect

**Figure 2:**
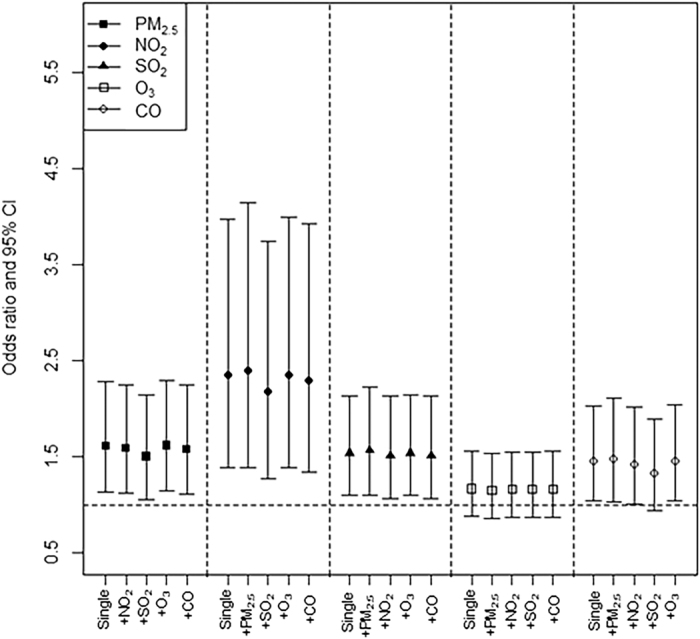
Odds ratios of Parkinson’s disease aggravation associated with a unit^a^ increase in the 8-day moving average (lag0–7) concentrations of 5 air pollutants: one-and two-pollutant models adjusted for the 2-day average (lag0–1) concentrations. ^a^Units are 10 μg/m^3^ for PM_10_; 10 ppb for NO_2_ and O_3_; 1 ppb for SO_2_; and 0.1 ppm for CO. ^b^The same-day (lag0) concentrations were used for O_3_.

According to the co-pollutant analyses, our estimated ORs showed consistent, significant associations after adjusting for the lag0–1 concentrations of other pollutants, except in the case of CO while controlling for SO_2_ (Fig. 2). The findings were similar following the adjustment for lag0 concentrations (Supplementary Fig. 4). Supplementary Fig. 5 presents the results of other sensitivity analyses. Generally, the estimated effects of air pollution based on the sensitivity analyses were smaller than those of the main analysis; however, they were still significant or showed trends with an identical direction as the main results.

## Discussion

In this time-stratified case-crossover study involving a representative national sample cohort, the risk of PD aggravation, defined as emergency hospital admission for primarily diagnosed PD, significantly increased during exposure to higher short-term concentrations of air pollutants. This association was constantly observed for PM_2.5_, NO_2_, SO_2_, and CO, and appeared to be robust to several sensitivity analyses. The group-specific results also showed a consistent pattern for each pollutant. To our knowledge, no study has explored the short-term associations between all 5 air pollutants and PD aggravation in humans, although the long-term associations have been evaluated previously^12^,^17^. Our findings suggest that short-term exposure to air pollution may also affect neurological disease progression.

Our estimated OR for PD aggravation associated with a 10 μg/m^3^ increase in the 2-day average concentrations of PM_2.5_ was 1.06 (95% CI: 0.89–1.26; 6.18%), which is larger than the estimate of the only available study, which reported a 3.23% increase in emergency admissions for PD in association with a 10 μg/m^3^ increase in the 2-day average concentrations^20^, although our result was not significant. The different effect estimates could be induced by various factors. Zanobetti *et al*.^20^ estimated the effect in the entire US; thus the exposure variability in the study is likely much larger than in Seoul. In particular, the authors included cities with missing daily exposure information if the cities have at least 219 days’ PM_2.5_ data in 1 year, which would likely affect the effect estimate^21^. In addition, Zanobetti *et al*.^20^ only adjusted for day of the week and temperature in the regression analysis and selected control days as every third day in the same month and year; in contrast, we adjusted for additional possible confounders and selected control days matched to the same day of the week within the same month and year. We also confirmed that the effect estimates with every third day as control days were lower than our main results in a sensitivity analysis. Another possible factor might be different particle composition. A previous study analyzed the chemical composition of PM_2.5_ in Seoul and compared the composition with that of the US^22^; the chemical composition of the US differed by regions and Seoul’s chemical structure (Higher NO_3_, Lower SO_4_) was similar to that of the western US, but different from that of the eastern US. Additionally, a different topography may explain the different result; the topography of Seoul is a basin surrounded by mountains, so the atmospheric diffusion was not easily achieved, causing a build-up of pollutants. This would likely affect the larger effect estimate in Seoul. Other possible factors that can contribute to the different effect estimates are difference in climate, which not only affect chemical reaction of particles but influence outdoor activity, and difference in distribution of potential modifiers of the association between PM_2.5_ and PD aggravation.

We consistently observed the strongest effect estimates for the 8-day moving average (lag0–7) concentrations for PM_2.5_, NO_2_, SO_2_, and CO. However, Zanobetti *et al*.^20^ reported a significant short-term association between PM_2.5_ and PD admissions only for the immediate lag (lag0, lag1, lag0–1, and lag0–2) concentrations, but not for the cumulative lags (lag0–4, and lag0–5). A recent prospective, community-based cohort study found a significant reduction in cerebral blood flow velocity in the middle cerebral artery, that was related to an increase in the averaged PM_2.5_ concentrations over the previous 1–28 days^19^. The study found stronger associations for more cumulative concentrations (7–, 14–, 21–, 28–day average) than immediate concentrations (1–, 3–day average), similar to our study. Moreover, previous studies identified stronger effect estimates for the cumulative (lag0–5) concentrations of air pollutants than for the immediate (lag0–1) concentrations in terms of cardiovascular and respiratory mortality^23^,^24^. Although it is likely that the cumulative concentrations of air pollutants have a stronger effect than the immediate concentrations on cardiovascular and respiratory diseases, future studies on short-term association between air pollution and neurological disease should investigate the effect of both immediate and cumulative concentrations, considering the inconsistent results between the previous studies.

The estimated ORs for gaseous pollutants were larger than that of PM_2.5_ when a same unit increase was applied. In particular, we found the strongest association between NO_2_ and PD aggravation. A possible reason for the larger effect of gaseous pollutants might be that the gaseous pollutants are likely better tracers of specific air pollution sources than PM_2.5_. For example, NO_2_ is generally considered a tracer of traffic emissions and SO_2_ is thought to be a tracer of power plant emissions. Particles, on the other hand, are more heterogeneous in origin, with multiple different sources contributing to their concentrations; therefore, if pollutants from a specific source are more biologically relevant, then tracers of that source would likely better capture the association. (e.g., the strongest association with NO_2_ might imply that traffic-related pollution is particularly important in this association, even if NO_2_ itself might not necessarily be the actual toxic agent). Although there has been no study on PD, the larger effect estimates of gaseous pollutants than particles were reported in studies on short-term association with non-accidental mortality and stroke mortality^25^,^26^. Further studies investigating association between neurological diseases and both gaseous pollutants and particles are warranted to establish more biologically relevant pollution sources.

Although each air pollutant has distinct physical or chemical characteristics, and multiple pathways are involved in disease initiation and progression, an inflammatory reaction is the common mechanism through which pollutants cause damage to human organs, including the CNS^6^,^27^; this mechanism may have contributed to the consistent results observed for each pollutant in our study. Recent research suggested that microglia—cells of the immune system in the brain—play an important role in this mechanism^6^. Air pollutants may directly activate microglia, or cytokines from the peripheral systemic inflammatory response can induce microglial activation. Microglial activation may lead to the development and aggravation of alpha-synucleinopathy^28^, a major component of PD pathogenesis. Additionally, changes in the BBB^29^ and the derivation of reactive oxygen species from air pollutants could induce the aggravation of CNS pathology^6^. Moreover, air pollution could indirectly influence emergency hospital admissions in PD patients with comorbidities associated with air pollution. For example, it has been reported that air pollution exposure aggravates respiratory disease, cardiovascular disease, and diabetes mellitus. These physical burdens could increase the risk of admission in PD patients^30^. However, the significant results of the association between air pollution and emergent admission in patients with primarily diagnosed PD supported the hypothesis that the effect of air pollution on the aggravation of movement symptoms was substantial.

We firstly investigated potential non-linear association between short-term exposure to all 5 air pollutants and PD aggravation, and no found evidence of non-linearity for any pollutants. Although the biological process and time scale of neurological disease are likely different from those for non-accidental mortality, it is quite agreed that there is no “safe” threshold level for air pollutants except O_3_ in short-term association with mortality^31^,^32^. However, it has been controversial whether the O_3_ and health association is linear^33^,^34^. Several experimental studies have found that O_3_ exposure causes BBB damage, dopamine neuron damage, and astrocyte death in the substantia nigra^9^,^35^. In particular, Zhou *et al*.^35^ found that only higher oxygen-O_3_ concentrations (60 μg/ml for 2 and 4 h) induced damage to astrocytes in rats, as compared with lower concentrations (20 and 40 μg/ml) *in vitro*; this finding suggests that a specific threshold of O_3_ may exist for neurological disease. The reason we did not find a significant O_3_ effect is likely because the O_3_ levels during the study period may have been below a threshold level that induces damage to human neurological system. Also, it is likely that we did not find evidence of non-linear association for O_3_, as our O_3_ levels were distributed below a threshold. Additionally, an O_3_ and mortality association study compared O_3_ levels and their effect estimates across various cities, and found that the relative risk was <1 when the level was <25 ppb, and was >1 when the level was >25 ppb^34^. Although threshold levels are likely different between outcome diseases, our O_3_ levels (24 ppb) during the case days would be below a threshold for PD aggravation.

The sex-, age-, and season-specific results were consistent for each pollutant; stronger effect estimates were observed in women, aged 65–74 years, and cold season in general, but the differences among group-specific results were not statistically significant. Our age-specific results showed statistically significant effects in PD patients aged 65–74 years but not in PD patients aged ≥75 years, even though the cases aged ≥75 years were predominant and aging is the key risk factor in PD^1^. We believe that PD patients aged ≥75 years in the present study likely stayed indoors, due to difficulty in movement as compared with younger patients. Therefore, their exposure to outdoor air pollution might be lower than that of younger patients. Moreover, the exposure measurement error would be greater in the elderly, because we assigned city-specific averages of concentrations instead of individual exposures to all patients, which could bias the observed effect estimate more towards the null, and appear as effect modification. Although lower effect estimates were observed among the elderly, the CIs widely overlapped with those of the other age groups. The CIs of sex- and season-specific effect estimates also overlapped and group-specific sample sizes may not have been sufficiently large to detect statistical significance in this study; therefore, drawing a conclusion about the effect modification would not be desirable. A few previous studies have examined effect modifications by potential modifiers in studying the relationship between air pollution and PD. Zanobetti *et al*.^36^ conducted an age-specific analysis and found a higher effect in subjects aged 65–74 years than subjects ≥75 years, but the difference was not significant in terms of the short-term association between PM_2.5_ and PD admissions. Liu *et al*.^14^ examined difference in sex-specific results in the long-term association between PM_10_ and PD, and reported a marginally significant higher effect in women (*p* interaction = 0.06). Although previous studies, including the present study, do not support significant effect modifications by potential modifiers, future studies examining effect modifiers may be informative and may contribute to elucidating potential biological mechanisms that can explain how air pollution affects PD.

The main results were robust to various sensitivity analyses. The effect estimates using alternate selection schemes for control days were slightly lower than those of main analysis. In the case-crossover analysis, selection of control period is one of the most important factors influencing effect estimation, and the results may differ markedly among selected controls. Several previous studies have proven that a time-stratified case-crossover design yields unbiased estimates among differently selected controls in air pollution epidemiology^37^,^38^. Similarly, our main result used the most finely time-stratified case-crossover design, where the control days were matched up to day of the week, and is therefore likely to be unbiased. When using different control selections, using every third day as control may have yielded lower effects as these days are too close to be independent, and temperature may not be a key confounder in the association of air pollution and PD; nevertheless, the results were relatively robust. The district-level analysis also showed positive associations with PD aggravation. While the estimated effect of PM_2.5_ was larger than that of the main analysis, the estimated effects of other pollutants were smaller than those of the main analysis. Additionally, the variation of PM_2.5_ effect increased and the effects of SO_2_ and CO became insignificant. This is likely due to missing information on district-specific averages, which causes biased estimates^21^ and reduced power due to deletion of cases with missing exposure data. The results restricting PD aggravation cases to patients’ first emergency admission showed the strongest association; including only data on the first admission of patients might better capture the association. However, the CIs of the results were considerably wide due to the smaller sample size (n = 57), and hence future studies are warranted. The estimated effects of NO_2_, SO_2_, and CO decreased when PD cases in 6 other metropolitan cities were included. This would likely be due to differences in topography and urban environment among cities; Seoul is located in a basin, whereas some cities are located in plains, where more atmospheric diffusion of pollutants is likely to occur than in a basin. Moreover, the population size (1,147,000–3,538,500) and the population density (1,022–4,452 individuals/km^2^) of other cities are much less than those of Seoul (size: 10,195,000 and density: 16,189 individuals/km^2^), and the chemical composition of air pollutants may differ according to the dominant industrial activities of the region. The main results conducted in Seoul would not be generalized to other cities or countries that have different characteristics; nevertheless, when combining other cities’ PD cases, the associations remained positive; this may indicate an association between short-term air pollution exposure and PD aggravation in other study areas. The analysis in which secondary PD admission cases were included revealed a positive, if less strong association. Although some diseases, such as cardiovascular and respiratory diseases, known to be related to air pollution exposure, may increase the emergency admissions of PD patients, some primary causes of secondary PD may be unrelated to air pollution; accordingly the effect estimates became smaller due to unrelated primary causes of emergency admissions.

Our study had certain limitations. First, we operationally defined PD aggravation as emergency admissions for primarily diagnosed PD without direct measurement of aggravation of PD symptoms, due to limited data availability. To be recognized as PD aggravations, two things must be met: (1) the patients in our study should already be PD patients, and (2) the primary cause for the emergency admission of PD patients included in the main analysis (77 cases) actually needed to be PD. For the first thing, we confirmed that all 314 cases (primary and accessory diagnosed PD) had have visited or hospitalized with diagnosed PD prior to the emergency admissions. For the second thing, we utilized the fact that in South Korea, PD is one of the rare intractable diseases for which the government pays 90% of the total medical treatment cost; however, the patients need to receive a definite diagnosis of PD based on imaging examinations, such as positron emission tomography-computed tomography (PET-CT) to be eligible as beneficiaries. Thus, we checked whether the government paid 90% of the treatment cost for the 77 primary PD cases. If the 77 cases were beneficiaries, then they would receive a definite diagnosis of PD by imaging examinations. We confirmed that almost every case only paid about 10% of the total treatment cost and every case paid less than 20% of the total cost, which is for ordinary inpatients without rare intractable diseases. Hence, the diagnosis of patients with PD in the NHIS-NSC was likely to be reliable. Second, our study population size seems small, as our PD cases were selected from the NHIS-NSC, comprising 2.2% of the total eligible population proportionally sampled in 18 age groups. However, there may be a larger number of PD patients at risk of developing PD aggravation due to air pollution in the entire population. Nevertheless, we found a consistent and significant association between short-term exposure to air pollution and PD aggravation by using a time-stratified, case-crossover analysis, in which each case had 3–4 controls; hence, the actual statistical analysis was conducted on a sample that was 3–4 times larger than the sample number. Such a case-crossover analysis is reportedly suitable for acute and rare outcomes, as it provides adequate statistical power^39^; therefore, our results are likely unbiased with regard to sample size. Third, there is a possibility of exposure measurement error, as our daily representative concentrations of air pollutants may not be represent of each individual’s actual exposure. Exposure measurement error may also occur if a family member who is not residing with the PD patient applied for medical insurance for the PD patient, as the residential address specified in the NHIS-NSC form would be that of the insurant. However, the measurement error in exposure estimates tends to cause bias towards a null hypothesis and would hence underestimate the association, rather than cause a false-positive result^40^; therefore, our results are not likely to be exaggerated.

The strength of our study is that it is the first study to examine the short-term effects of 5 air pollutants on PD aggravation in humans, in a representative, population-based cohort. The NHIS-NSC provides comprehensive and detailed information on medical care utilization of citizens, based on claims data reflecting actual clinical practice. Thus, only the PD cases who received medical treatment in hospitals were included in our study. Although the exact pathogenesis of PD remains unclear, many risk factors, such as occupational exposure, smoking, coffee consumption, and genetic risk factors, have been suggested to be related to PD incidence and prevalence^1^. We controlled for these individual confounding factors by using a case-crossover design with perfect matching, and confirmed the independent association between air pollution and PD aggravation. Moreover, we assessed potential effect modification by sex, age, and season, and these findings could contribute to the creation of public health policy for the prevention of PD deterioration by providing information on the highly susceptible group.

Overall, the findings of this study, involving a representative population-based cohort, suggest that short-term exposure to air pollution may increase the risk of PD aggravation. Our results can serve as the basis for further studies on the short-term association between air pollution and neurological diseases, and for policy-making to mitigate air pollution and reduce neurodegenerative health effects in our aging society.

## Methods

### Study population

The National Health Insurance Service (NHIS) is a health insurance system with universal coverage in Korea, in which all citizens are registered. The NHIS established the National Health Information Database (NHID) that contains individual information, demographic data, and medical treatment information of Korean citizens since its formation in 2000. In 2015, the NHIS released data of the National Health Insurance Service-National Sample Cohort (NHIS-NSC), which is a representative, population-based cohort proportionally stratified by age, sex, and income level. The cohort includes 1,025,340 patients sampled from the target population of 46,605,433 individuals in the 2002 NHID, who were followed until 2013. Detailed information on the NHIS-NSC has been described elsewhere^41^.

We obtained information on our study population from NHIS-NSC data for 2002 through 2013. We used information on patient demographics (sex, age, and district-level residential address) and medical treatment (date of visit, primary and accessory diagnosis, type of medical department, type of visits [outpatient or inpatient], and admission route). Before outcome ascertainment, we operationally defined PD aggravation, based on previous studies^17^,^20^, as cases of emergency admission wherein PD was primarily diagnosed. We first collected data on the medical treatment of patients diagnosed with PD (International Classification of Disease, 10^th^ Revision code G20 for primary or accessory diagnosis) in 7 metropolitan cities in Korea (n = 191,012). Thereafter, we only assessed admission cases (n = 43,071). Among the admission cases, we only selected patients residing in Seoul (n = 14,774). Seoul was chosen as the study area for our main analysis because regular measurement of PM_2.5_ has been conducted only in Seoul. Furthermore, it is a suitable study location, considering the dense population and the heavy traffic; Seoul’ population accounts for one-fifth of the entire population of Korea and its population density is 16,189 individuals/km^2^. Finally, among the admission cases in Seoul, only emergency admission cases (n = 314) were selected, as scheduled admissions would not have been appropriate for assessing the short-term association (Supplementary Fig. 1).

### Ethics statement

Ethical approval was obtained from the Institutional Review Board of Seoul National University (Approval Number: 1410/002-009). All methods were performed in accordance with the relevant guidelines and regulations. The NHIS provided the data after encryption to protect private information. Therefore, the need for informed consent was waived.

### Air pollution and weather information

Seoul is composed of 25 districts ranging between 10 to 47 km^2^. We obtained hourly concentrations of nitrogen dioxide (NO_2_), sulfur dioxide (SO_2_), ozone (O_3_), and carbon monoxide (CO) from 27 monitoring sites operated by the Korean National Institute of Environmental Research, as well as those of particles <2.5 μm (PM_2.5_) from 25 monitoring sites operated by the Seoul Research Institute of Public Health and Environment (only at 7 sites in 2002, which expanded gradually to 25) in Seoul. Each district has at least 1 monitoring site. During study period, there were substantial missing daily concentrations when daily exposure concentrations were constructed for each monitoring site, as mechanical failure at a monitoring site usually persists for a day (range: 18–1,822 missing values over a total of 4,383 days). Analysis with missing data can cause biased estimates^21^ and reduced power due to deletion of cases with missing exposures. To reduce the bias attributed to missing exposures, we constructed daily representative concentrations as follows: first, we calculated the mean hourly concentrations by averaging the concentrations measured at each monitoring site. This method appropriately resolved the missing hourly values, as none of the cases had simultaneously missing hourly values across different sites at a certain hour. Second, the 24-h concentrations were averaged for PM_2.5_, NO_2_, and SO_2_, whereas the daytime 8-h [09:00–17:00] concentrations were averaged for O_3_ and CO. We chose daytime average of O_3_ and CO concentrations to represent outdoor exposures better, considering that people are more likely to be active outdoors during the daytime. These city-specific concentrations as measures of short-term exposures solve the bias created by missing exposures. Although assigning averaged exposures measured via fixed monitoring sites to each individual causes a Berkson error, the error causes little or no bias in measurement and thus in risk estimates^42^.

Hourly data on ambient temperature, relative humidity, and air pressure were obtained from the Korea Meteorological Administration; daily mean values were constructed to adjust for potential confounding by short-term variations in weather variables.

### Study design

We applied a time-stratified case-crossover design to explore the short-term association between air pollution and PD aggravation. A case-crossover design is a variant of the case-control design for the evaluation of transient exposures and rare acute-onset diseases that provides sufficient statistical power for fewer cases^39^. As each patient serves as his own control, time-invariant individual factors, such as sex and genetic predisposition, are automatically controlled by perfect matching. Factors that vary gradually, such as seasonal patterns and long-term trends, can be controlled by choosing control days that are close to the case days^43^. We selected 3 or 4 control days matched to the same day of the week within the same month and year as the admission day (case day) for each case (e.g., if admission occurred on a Monday in March 2002, then the remaining Mondays in that month were chosen as the control days). This time-stratified design has been shown to yield unbiased effect estimates, with an adequate conditional logistic likelihood^38^.

### Statistical analysis

The odds ratios (ORs) and 95% confidence intervals (CIs) for the risk of PD aggravation, associated with short-term air pollution exposure, were estimated by using conditional logistic regression, which compares the air pollutants concentrations on the case and control days. We adjusted for moving average temperature of the same day and previous day (lag0–1) by using a regression spline (with 3 df) to control for potential nonlinear associations, and adjusted for relative humidity and air pressure on same day (lag0), influenza epidemics, holidays, and the first day of the month; the final adjustment was made to reduce bias due to case misclassification, as the information of long-term in-patients is additionally recorded on every first day of each month because the NHIS provider submits claims to the Health Insurance Review Agency on the first day.

We entered each air pollutant linearly into the model with various lag structures in order to consider the immediate, delayed, and cumulative effects (single lag: same day and 1–7 days before admission [lag0 to lag7]; moving average lag: moving average of same day and 1–7 days before admission [lag0–1 to lag0–7]), and plotted the effect estimates of each pollutant at all lag structures to explore the lag pattern for different exposures. Among the lag structures, the lag that derived the strongest effect estimates was chosen as the main exposure for each air pollutant. This method has been recognized as an appropriate strategy for selecting lag structures when evaluating different exposures and outcome associations^23^,^24^. For each pollutant, we tested for possible non-linearity in association with PD aggravation using a restricted natural cubic spline function with 3 knots^44^. The placement of knots was selected based both on the distribution and air quality guidelines^45^,^46^. The test for non-linearity is equivalent to testing the significance of the spline.

In the case-crossover design, the effects of time-invariant factors cannot be estimated due to perfect matching. We examined the potential effect modification by sex, age (<65 years, 65–74 years, ≥75 years), and seasons (warm: April–September, cool: October–March), using the interaction terms of the modifiers and air pollutants maintaining statistical power.

### Sensitivity analysis

We conducted several sensitivity analyses. First, we conducted co-pollutant analyses to investigate potential confounding by other pollutants. For the co-pollutants, both same-day (lag0) and 2-day average (lag0–1) concentrations were analyzed. Second, we tested alternate selection schemes for control days: a) every third day in the same month and year as the case day, and b) temperature-matched day in the same month and year as the case day (temperature rounded to the nearest degree in °C). The day of the week was further controlled in the alternate control models. Third, we conducted a district-level analysis by assigning district-specific averages to patients in order to investigate how the results using district-specific concentrations with missing values are different. Fourth, we only included patients’ first admission case to confirm the sensitivity of the results to allow for multiple emergent admissions. Fifth, we included patients residing in the other 6 metropolitan cities (Incheon, Busan, Daegu, Daejeon, Gwangju, and Ulsan) in our study population for generalizability. We assigned city-level exposure variables to each patient and analyzed without any model stratification by city due to the small number of cases per city (range: 2–28). Lastly, we defined PD aggravation as either a primary or an accessory diagnosis.

All analyses were conducted using the PHREG method in SAS 9.4 (SAS Institute, Cary, NC). The results were reported as estimated ORs with 95% CIs per a unit increase in the concentrations of the 5 air pollutants; units are 10 μg/m^3^ for PM_10_; 10 ppb for NO_2_ and O_3_; 1 ppb for SO_2_; and 0.1 ppm for CO.

## Additional Information

**How to cite this article:** Lee, H. *et al*. Short-term air pollution exposure aggravates Parkinson’s disease in a population-based cohort. *Sci. Rep.* **7**, 44741; doi: 10.1038/srep44741 (2017).

**Publisher's note:** Springer Nature remains neutral with regard to jurisdictional claims in published maps and institutional affiliations.

## Supplementary Material

Supplementary Information

## Figures and Tables

**Table 1:** Cases of emergency hospital admissions for Parkinson’s disease (PD): Overall and according to sex, age, season, and comorbidity in the NHIS-NSC, Seoul.

Variables	Primary PD diagnosis		Primary and accessory PD diagnosis	
	*n*	%	*n*	%
Overall	77		314	
Sex				
Male	34	44	151	48
Female	43	56	163	52
Age				
≤64	8	10	34	13
65–74	28	37	114	36
≥75	41	53	159	51
Season				
Warm (April–September)	36	47	159	51
Cool (October–March)	41	53	155	49
Accessory diagnosis				
Dementia	9	12		
Diabetes	9	12		
Cerebral infarction	7	9		
Primary diagnosis				
Respiratory			40	13
Cerebrovascular			33	11
Genitourinary			24	8

**Table 2:** Odds ratios of Parkinson’s disease aggravation associated with a unit^a^ increase in the 8-day moving average (lag0–7) concentrations of 5 air pollutants: effect modification by age, sex, and season.

	PM_2.5_		NO_2_		SO_2_		O_3_^c^		CO	
	OR (95% CI)	*p value*^*b*^	OR (95% CI)	*p value*	OR (95% CI)	*p value*	OR (95% CI)	*p value*	OR (95% CI)	*p value*
All	1.61 (1.14, 2.29)		2.35 (1.39, 3.97)		1.54 (1.11, 2.14)		1.17 (0.88, 1.55)		1.46 (1.05, 2.04)	
Sex										
Male	1.53 (0.86, 2.72)	0.82	2.81 (1.17, 6.76)	0.61	1.46 (0.94, 2.25)	0.72	1.08 (0.71, 1.65)	0.64	1.34 (0.83, 2.15)	0.62
Female	1.66 (1.10, 2.50)		2.15 (1.16, 3.99)		1.64 (1.02, 2.63)		1.23 (0.86, 1.78)		1.56 (1.02, 2.37)	
Age										
≤64	1.63 (0.66, 4.04)	0.58	3.15 (0.59, 16.9)	0.50	1.55 (0.70, 3.44)	0.99	1.10 (0.44, 2.77)	0.23	1.15 (0.59, 2.24)	0.60
65–74	1.95 (1.14, 3.32)		2.62 (1.27, 5.40)		1.49 (0.88, 2.52)		0.80 (0.47, 1.36)		1.74 (1.04, 2.91)	
≥75	1.32 (0.78, 2.24)		1.9 (0.86, 4.21)		1.57 (0.99, 2.50)		1.38 (0.96, 1.98)		1.39 (0.86, 2.26)	
Season										
Warm	1.34 (0.81, 2.23)	0.36	1.86 (0.48, 4.11)	0.45	1.27 (0.73, 2.21)	0.42	1.24 (0.90, 1.70)	0.39	1.38 (0.69, 2.77)	0.86
Cool	1.83 (1.17, 2.87)		2.77 (1.38, 5.57)		1.68 (1.13, 2.51)		0.92 (0.49, 1.70)		1.48 (1.02, 2.16)	

^a^Units are 10 μg/m^3^ for PM_10_; 10 ppb for NO_2_ and O_3_; 1 ppb for SO_2_; and 0.1 ppm for CO.

^b^*p* value for the difference in the estimated effects of pollutants on the risk of Parkinson’s disease aggravation between sex-, age-, and season-specific associations.

^c^The same-day (lag0) concentrations were used for O_3_.
