# Supplementary material for: Short-term air pollution exposure aggravates Parkinson’s disease in a population-based cohort
Source: Sci Rep. 2017 Mar 16;7:44741. doi: 10.1038/srep44741 (PMC5353743; doi:10.1038/srep44741)
Supplement: Supplementary Information [file srep44741-s1.doc]

**Supplementary Information**

**Short-term air pollution exposure aggravates Parkinson’s disease in a population-based cohort**

Hyewon Lee^1^, Woojae Myung^2^, Doh Kwan Kim^2^, Satbyul Estella Kim^1^, Clara Tammy Kim^1^, and Ho Kim^1,*^

^1^Graduate School of Public Health, Seoul National University, South Korea; ^2^Department of Psychiatry, Samsung Medical Center, Sungkyunkwan University School of Medicine, Seoul, South Korea

E-mail addresses: [woniggo@gmail.com](mailto:woniggo@gmail.com) (H. Lee), [hokim@snu.ac.kr](mailto:hokim@snu.ac.kr) (H. Kim).

**Supplementary Table 1.** Distribution (mean±standard deviation [SD]/interquartile range [IQR]) of the environmental variables during the case and control study periods.

|  | Primary PD diagnosis | | | | | | Primary and accessory PD diagnosis | | | | | |
| --- | --- | --- | --- | --- | --- | --- | --- | --- | --- | --- | --- | --- |
|  | Case | | | Control | | | Case | | | Control | | |
| **Variables** | N | Mean ± SD | IQR | N | Mean ± SD | IQR | N | Mean ± SD | IQR | N | Mean ± SD | IQR |
| PM_2.5_(µg/m^3^)^a^ | 77 | 27.4 ± 12.7 | 16.6 | 268 | 27.5 ± 17.9 | 16.5 | 314 | 26.3 ± 14.1 | 15.3 | 1084 | 26.7 ± 15.3 | 17.4 |
| NO_2_(ppb)^a^ | 77 | 36.2 ± 11.5 | 15.3 | 268 | 36.1 ± 11 | 16.5 | 314 | 36.1 ± 11.1 | 15.2 | 1084 | 35.9 ± 11 | 16.1 |
| SO_2_(ppb)^a^ | 77 | 5.7 ± 2.6 | 1.9 | 268 | 5.3 ± 1.9 | 2.8 | 314 | 5.3 ± 2.1 | 2.2 | 1084 | 5.2 ± 2 | 2.5 |
| O_3_(ppb)^a^ | 77 | 24.1 ± 12.4 | 16.3 | 268 | 22.9 ± 11.3 | 15.8 | 314 | 24.8 ± 12.6 | 17.5 | 1084 | 23.9 ± 12 | 17 |
| CO(ppm)^a^ | 77 | 0.58 ± 0.22 | 0.2 | 268 | 0.57 ± 0.21 | 0.25 | 314 | 0.55 ± 0.21 | 0.21 | 1084 | 0.56 ± 0.22 | 0.24 |
| PM_2.5_(µg/m^3^)^b^ | 76 | 28.7 ± 14.9 | 13.5 | 268 | 26.4 ± 10.5 | 12.6 | 313 | 27.7 ± 12.4 | 13.8 | 1083 | 26.6 ± 10.4 | 12.4 |
| NO_2_(ppb)^b^ | 76 | 37.2 ± 9.4 | 13.1 | 268 | 35.6 ± 7.5 | 11.3 | 313 | 36.4 ± 8.2 | 10.1 | 1083 | 35.8 ± 7.6 | 10.6 |
| SO_2_(ppb)^b^ | 76 | 5.6 ± 2 | 3 | 268 | 5.3 ± 1.6 | 2.6 | 313 | 5.4 ± 1.7 | 2.3 | 1083 | 5.3 ± 1.7 | 2.2 |
| O_3_(ppb)^b^ | 76 | 23 ± 10.1 | 15.4 | 268 | 23.2 ± 9.9 | 15.2 | 313 | 24.1 ± 10.4 | 16.5 | 1083 | 24.2 ± 10.4 | 16.7 |
| CO(ppm)^b^ | 76 | 0.58 ± 0.18 | 0.25 | 268 | 0.57 ± 0.17 | 0.22 | 313 | 0.56 ± 0.17 | 0.21 | 1083 | 0.56 ± 0.17 | 0.2 |
| Temperature(℃) | 77 | 11.5 ± 12.1 | 23.1 | 268 | 12.1 ± 11.1 | 19.8 | 314 | 12.9 ± 11.1 | 18.9 | 1084 | 12.8 ± 10.5 | 18.6 |
| Humidity(%) | 77 | 48.4 ± 21.1 | 29.2 | 268 | 53 ± 22.9 | 30.3 | 314 | 50.3 ± 19.8 | 28.4 | 1084 | 52.9 ± 21 | 30.3 |
| Air pressure(hPa) | 77 | 766.8 ± 440.1 | 20.3 | 268 | 774.4 ± 433.1 | 19.9 | 314 | 764.7 ± 438.7 | 21 | 1084 | 771.2 ± 434 | 22.1 |

^a^The 2-day moving average (lag0–1) concentrations are described. ^b^The 8-day moving average (lag0–7) concentrations are described.

**Supplementary Table 2.** Correlations between exposure variables in Seoul, Korea, during 2002–2013.

|  | PM_2.5_ | NO_2_ | SO_2_ | O_3_ | CO | Temperature | Humidity | | Air pressure |
| --- | --- | --- | --- | --- | --- | --- | --- | --- | --- |
| PM_2.5_ | 1.00 | 0.56* | 0.54* | 0.00 | 0.60* | -0.08* | 0.10* | 0.13* | |
| NO_2_ |  | 1.00 | 0.69* | -0.2* | 0.76* | -0.23* | -0.12* | 0.02 | |
| SO_2_ |  |  | 1.00 | -0.19* | 0.74* | -0.44* | -0.19* | 0.00 | |
| O_3_ |  |  |  | 1.00 | -0.44* | 0.47* | -0.15* | -0.07* | |
| CO |  |  |  |  | 1.00 | -0.43* | 0.02 | 0.05* | |
| Temperature |  |  |  |  |  | 1.00 | 0.36* | 0.00 | |
| Humidity |  |  |  |  |  |  | 1.00 | 0.68* | |
| Air pressure |  |  |  |  |  |  |  | 1.00 | |

*Statistically significant correlation is indicated by *p*<0.05.

**Supplementary Table 3.** Odds ratios of Parkinson’s disease aggravation associated with a unit^a^ increase in the 3-day lagged concentrations of air pollutants: effect modification by age, sex, and season.

|  | PM_2.5_ | | NO_2_ | | SO_2_ | | O_3_ | | CO | |
| --- | --- | --- | --- | --- | --- | --- | --- | --- | --- | --- |
|  | OR (95% CI) | *p value^b^* | OR (95% CI) | *p value* | OR (95% CI) | *p value* | OR (95% CI) | *p value* | OR (95% CI) | *p value* |
| All | 1.29 (1.06, 1.57) |  | 1.55 (1.19, 2.03) |  | 1.15 (0.96, 1.39) |  | 0.77 (0.57, 1.05) |  | 1.22 (1.05, 1.41) |  |
| Sex |  |  |  |  |  |  |  |  |  |  |
| Male | 1.10 (0.82, 1.47) | 0.13 | 1.39 (0.92, 2.10) | 0.49 | 0.94 (0.72, 1.22) | 0.02 | 1.02 (0.63, 1.65) | 0.14 | 1.12 (0.9, 1.39) | 0.30 |
| Female | 1.48 (1.13, 1.94) |  | 1.68 (1.18, 2.37) |  | 1.49 (1.11, 2.00) |  | 0.63 (0.41, 0.97) |  | 1.31 (1.07, 1.61) |  |
| Age |  |  |  |  |  |  |  |  |  |  |
| ≤64 | 1.10 (0.69, 1.75) | 0.32 | 2.42 (0.89, 6.62) | 0.24 | 1.00 (0.63, 1.60) | 0.46 | 0.69 (0.26, 1.82) | 0.87 | 1.25 (0.86, 1.83) | 0.11 |
| 65–74 | 1.57 (1.13, 2.17) |  | 1.92 (1.21, 3.07) |  | 1.41 (0.96, 2.08) |  | 0.85 (0.52, 1.40) |  | 1.61 (1.17, 2.21) |  |
| ≥75 | 1.18 (0.89, 1.56) |  | 1.27 (0.47, 3.31) |  | 1.10 (0.86, 1.41) |  | 0.73 (0.47, 1.12) |  | 1.09 (0.90, 1.31) |  |
| Season |  |  |  |  |  |  |  |  |  |  |
| Warm | 1.14 (0.82, 1.57) | 0.32 | 1.41 (0.87, 2.27) | 0.64 | 1.15 (0.80, 1.66) | 0.98 | 0.92 (0.65, 1.31) | 0.05 | 1.26 (0.91, 1.75) | 0.82 |
| Cool | 1.39 (1.09, 1.78) |  | 1.62 (1.18, 2.22) |  | 1.16 (0.93, 1.44) |  | 0.42 (0.21, 0.84) |  | 1.21 (1.02, 1.43) |  |

^a^Units are 10 μg/m^3^ for PM_10_; 10 ppb for NO_2_ and O_3_; 1 ppb for SO_2_; and 0.1 ppm for CO.

^b^*p* value for the difference in the estimated effects of pollutants on the risk of Parkinson’s disease aggravation between sex-, age-, and season-specific associations.

**Supplementary Figure 1.** Flow chart of the study population from the NHIS-NSC, Seoul, Korea, 2002-2013.


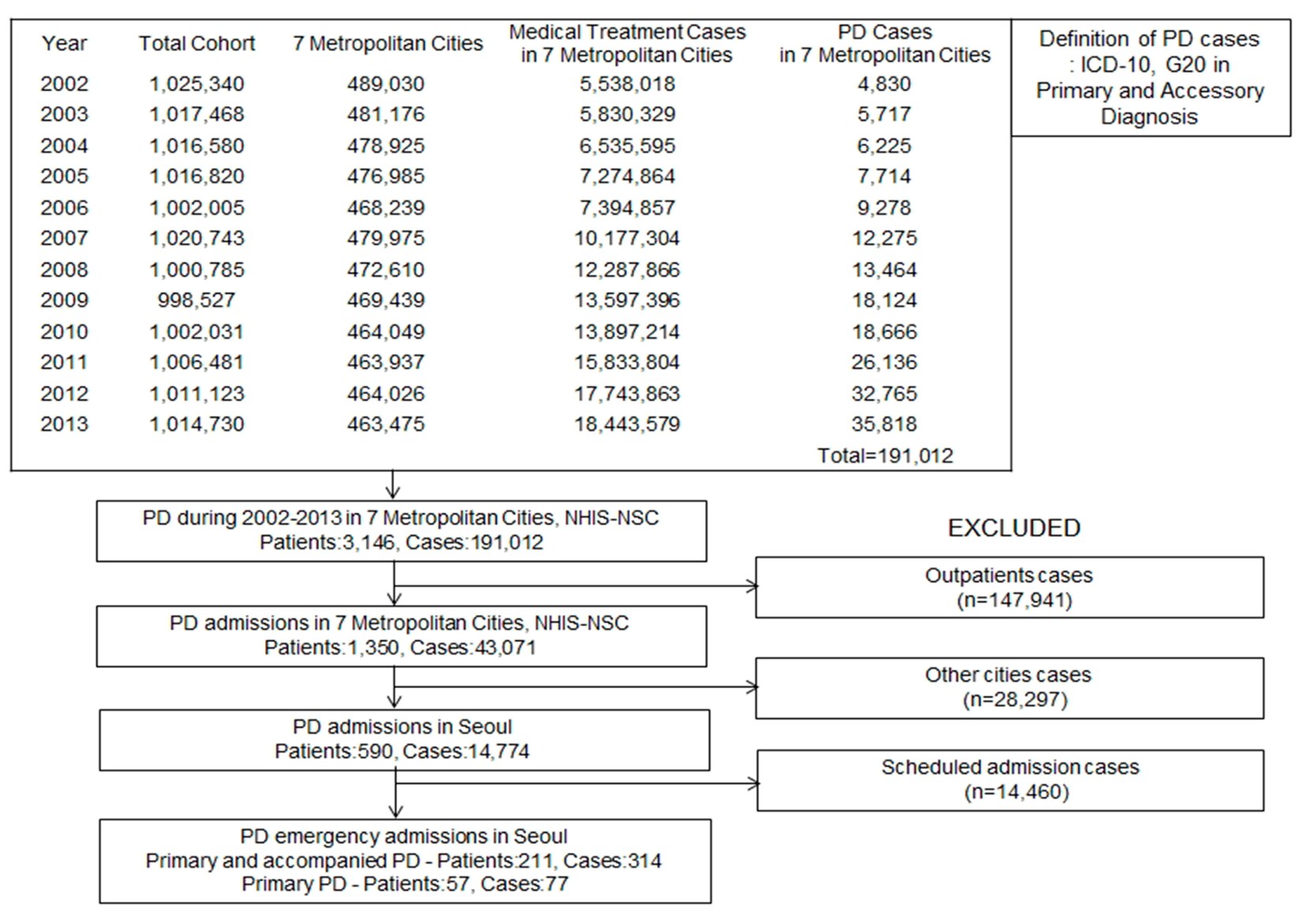


**Supplementary Figure 2.** Odds ratios of emergency hospital admissions with Parkinson’s disease as a primary or an accessory diagnosis associated with a unit^a^ increase in the concentrations of 5 air pollutants with various lag structures (single lag on the same day [lag0] and on the previous 1–7 days [lag1–lag7], as well as moving average lag on the same day plus 1 day before [lag0–1] to 7 days before [lag0–7]).


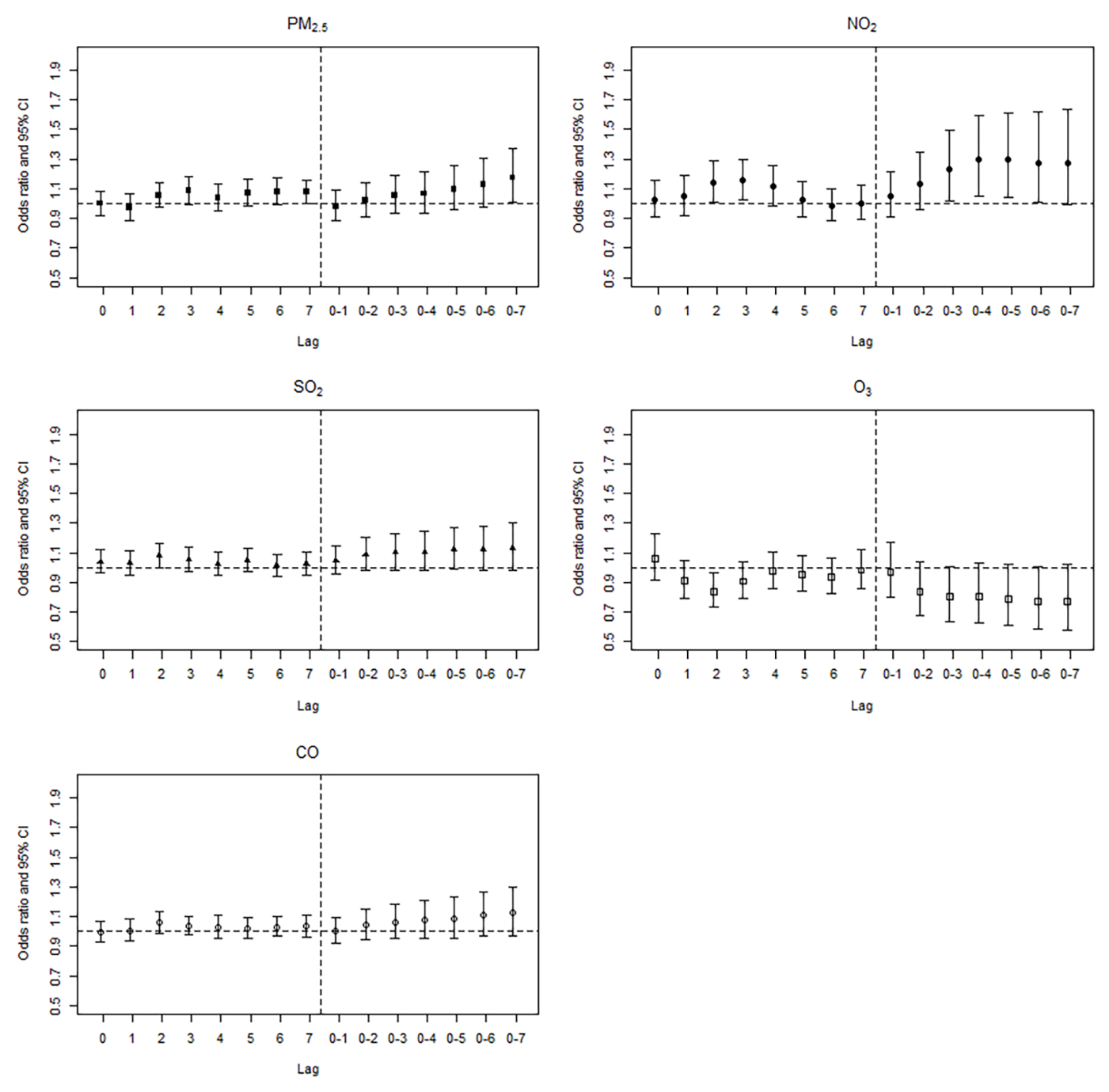


^a^Units are 10 μg/m^3^ for PM_10_; 10 ppb for NO_2_ and O_3_; 1 ppb for SO_2_; and 0.1 ppm for CO.

**Supplementary Figure 3.** Concentration-response associations between air pollutants and Parkinson’s disease aggravation using restricted cubic splines with 3 knots.


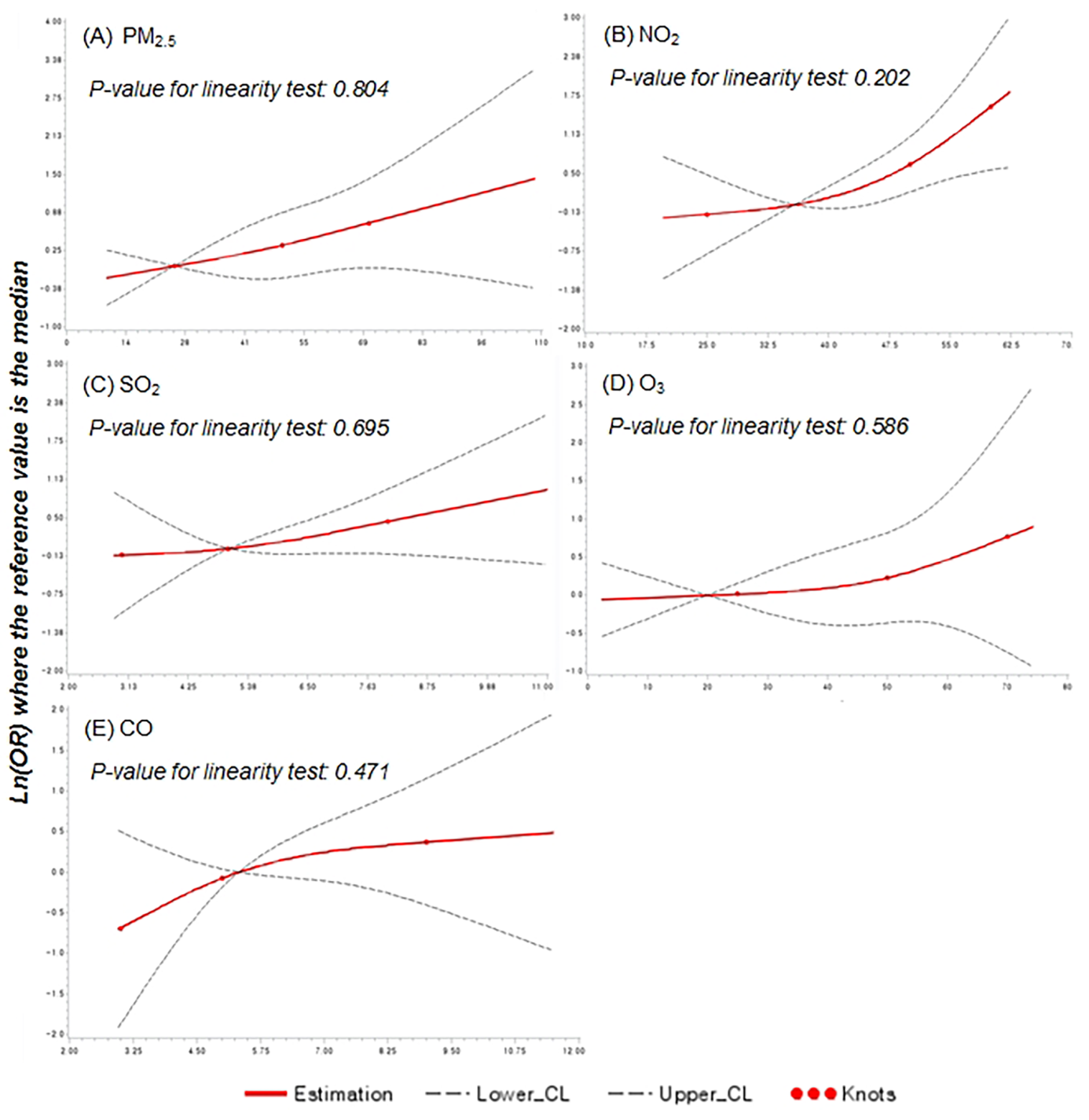


^a^Knots were located at 25, 50, and 70 µg/m^3^ for a 24-h average of PM_2.5_; at 20, 40, and 60 ppb for a 24-h average of NO_2_; at 3, 5, and 8 ppb for a 24-h average of SO_2_; at 25, 50, and 70 ppb for an 8-h average of O_3_; and at 0.3, 0.5, and 0.9 ppm for an 8-h average of CO. ^b^Temperature, relative humidity, air pressure, influenza epidemics, holidays, and the first day of the month were adjusted. ^c^The lag0–7 concentrations of all the pollutants, except for O_3_, were analyzed (lag0 concentrations).

**Supplementary Figure 4.** Odds ratios of Parkinson’s disease aggravation associated with a unit^a^ increase in the 8-day moving average (lag0–7) the concentrations of 5 air pollutants: one-and two-pollutant models adjusted for same day (lag0) concentrations


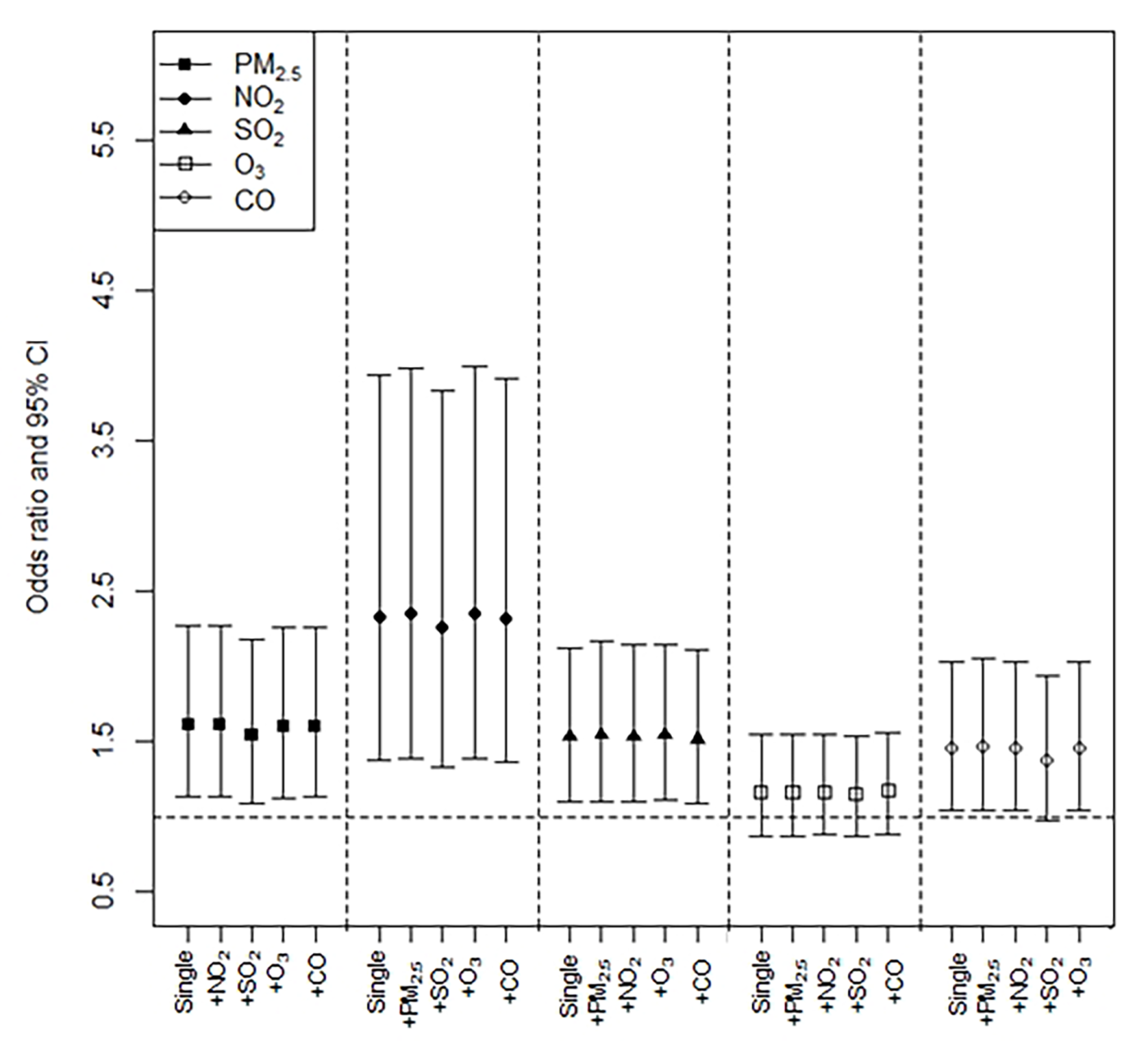


^a^Units are 10 μg/m^3^ for PM_10_; 10 ppb for NO_2_ and O_3_; 1 ppb for SO_2_; and 0.1 ppm for CO. ^b^The same-day (lag0) concentrations were used for O_3_.

**Supplementary Figure 5.** Odds ratios (95% confidence intervals) for emergency admissions for Parkinson’s disease, associated with a unit^a^ increase in the 8-day moving average (lag0–7) the concentrations of 5 urban air pollutants: results of the main models and sensitivity analyses.


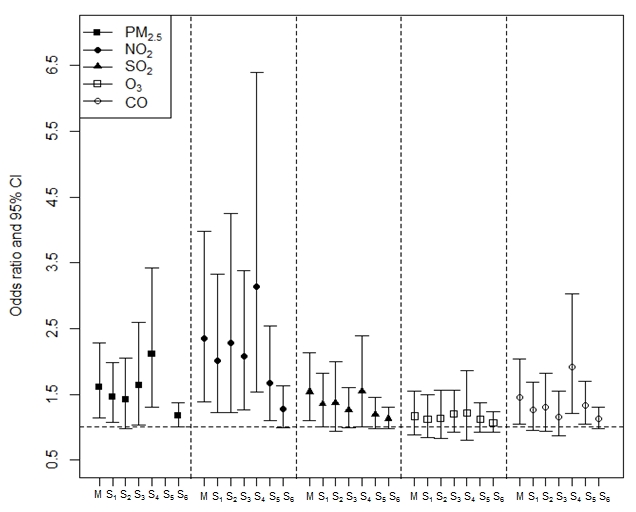


^a^Units are 10 μg/m^3^ for PM_10_; 10 ppb for NO_2_ and O_3_; 1 ppb for SO_2_; and 0.1 ppm for CO. ^b^The same-day (lag0) concentrations were used for O_3_. ^c^The abbreviations of sensitivity analyses: M, main results; S_1_, control chosen as every 3^rd^ day; S_2_, control chosen as a temperature-matched day; S_3_, use of district-level concentrations; S_4_, restriction of PD cases to patients’ first emergent admissions; S_5_, inclusion of the PD cases of 6 other metropolitan cities; and S_6_, inclusion of patients with PD as the accompanying disease. ^d^S_5_ was not analyzed for PM_2.5_ due to the unavailability of data.
